# Supplementary figures and images for: The Mediating Role of Psychological Resilience in the Relationship between Emotional Reactivity, Intolerance of Uncertainty and Psychological Maladjustment in Children Receiving Orthodontic Treatment
Source: Healthcare (Basel). 2022 Aug 10;10(8):1505. doi: 10.3390/healthcare10081505 (PMC9408414; doi:10.3390/healthcare10081505)

## Supplementary Material

### 1.1 Supplementary Figures

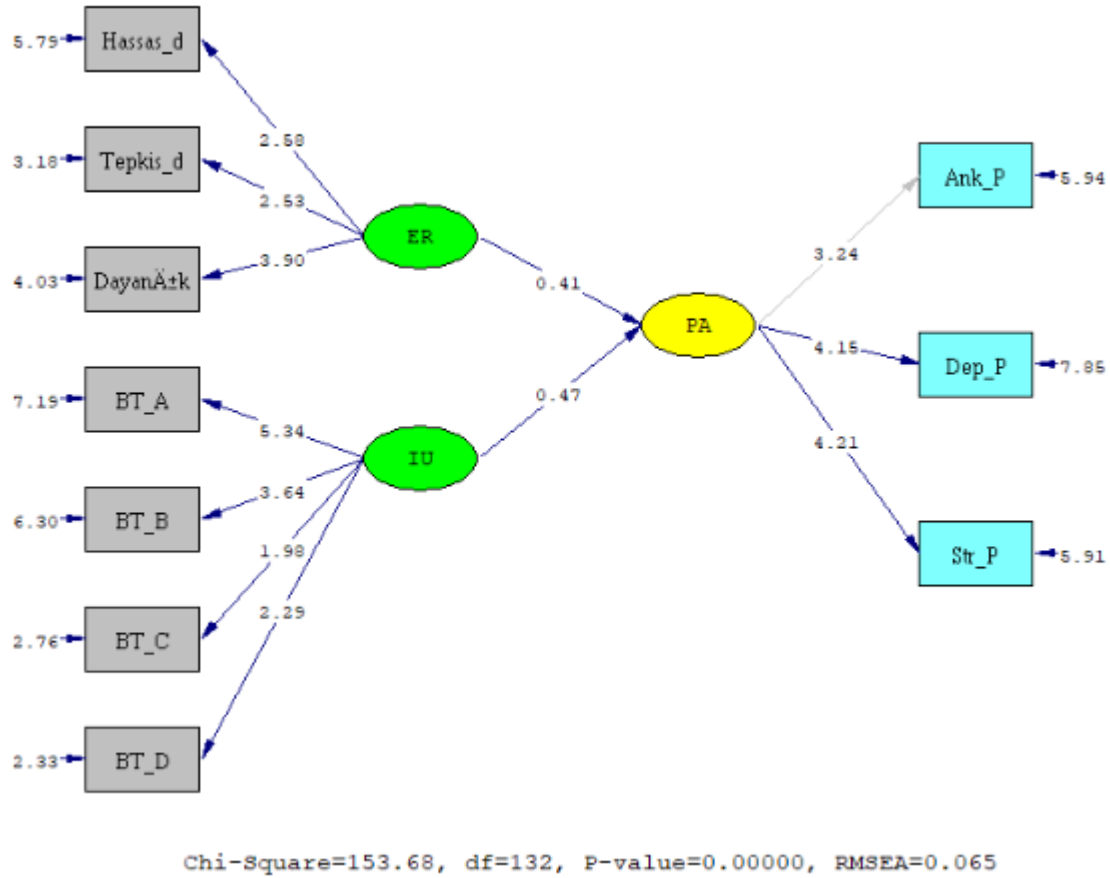

Figure S1. Model 1

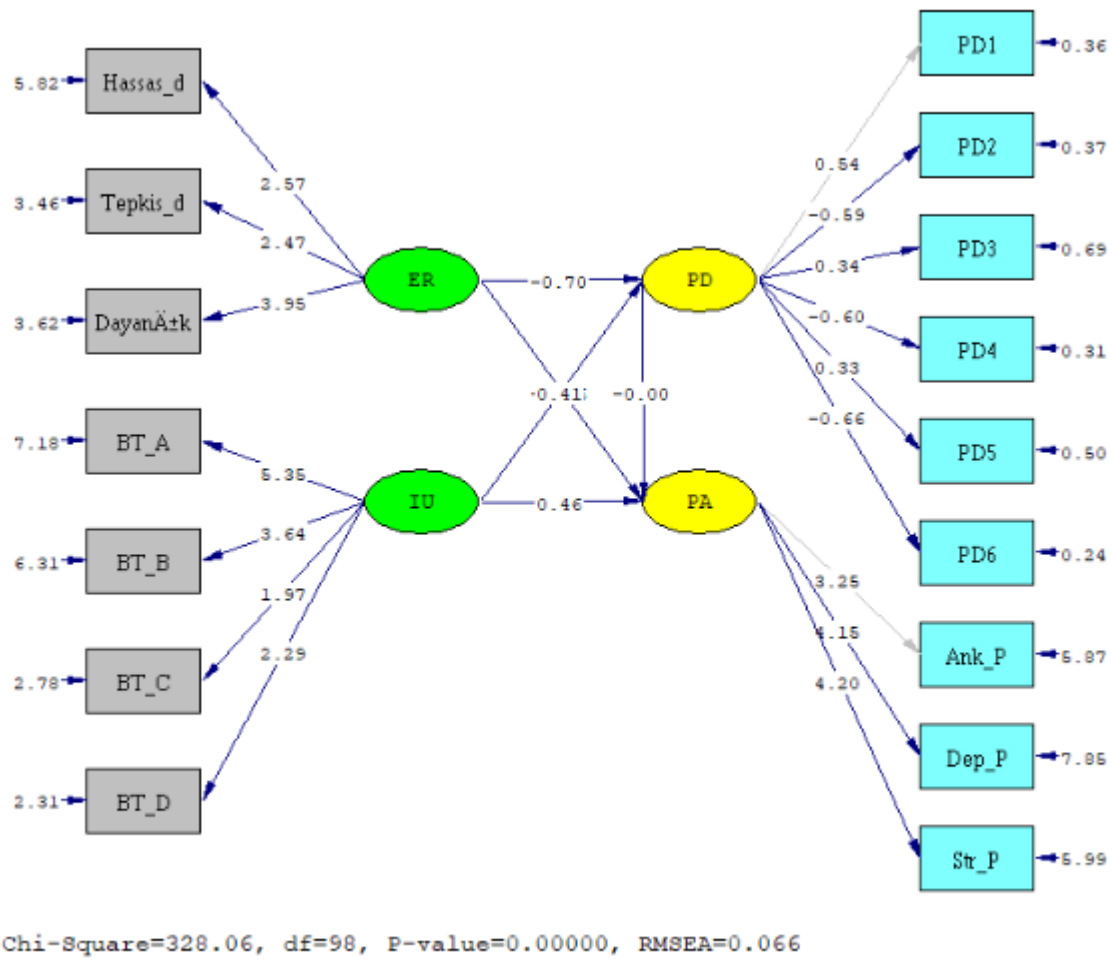

Figure S2. Model 2

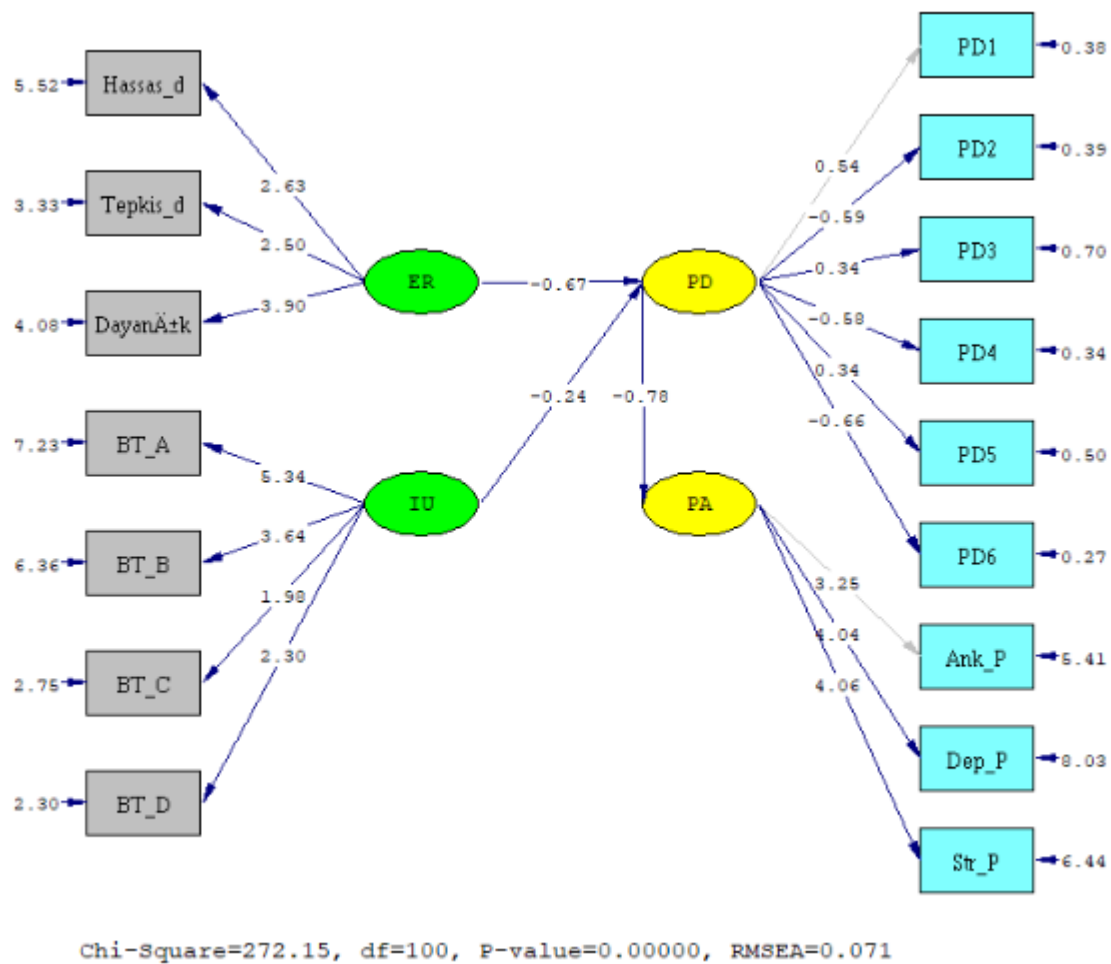

**Figure S3. Model 3**

Supplement: Supplementary file 1 [file healthcare-10-01505-s001.zip › healthcare-1775086-supplementary.pdf]
